# ER Stress-Sensor Proteins and ER-Mitochondrial Crosstalk—Signaling Beyond (ER) Stress Response

**DOI:** 10.3390/biom11020173

**Published:** 2021-01-28

**Authors:** Vaishali Kumar, Shuvadeep Maity

**Affiliations:** Department of Biological Sciences, Birla Institute of Technology and Science (BITS)-Pilani (Hyderabad Campus), Shameerpet-Mandal, Hyderabad, Telangana 500078, India; p20200006@hyderabad.bits-pilani.ac.in

**Keywords:** ER stress, endoplasmic reticulum, mitochondria associated membrane (MAM)

## Abstract

Recent studies undoubtedly show the importance of inter organellar connections to maintain cellular homeostasis. In normal physiological conditions or in the presence of cellular and environmental stress, each organelle responds alone or in coordination to maintain cellular function. The Endoplasmic reticulum (ER) and mitochondria are two important organelles with very specialized structural and functional properties. These two organelles are physically connected through very specialized proteins in the region called the mitochondria-associated ER membrane (MAM). The molecular foundation of this relationship is complex and involves not only ion homeostasis through the shuttling of calcium but also many structural and apoptotic proteins. IRE1alpha and PERK are known for their canonical function as an ER stress sensor controlling unfolded protein response during ER stress. The presence of these transmembrane proteins at the MAM indicates its potential involvement in other biological functions beyond ER stress signaling. Many recent studies have now focused on the non-canonical function of these sensors. In this review, we will focus on ER mitochondrial interdependence with special emphasis on the non-canonical role of ER stress sensors beyond ER stress.

## 1. Introduction

Eukaryotic cells have different membrane-bound compartments or organelle with specific biochemical and biological functions. Initially, to understand cellular homeostasis, understanding a particular organelle and their function was the main focus. In recent days, the crosstalk between different organelles is studied to elucidate their impact on various human diseases [1,2,3]. Inter-organellar connections are achieved by direct physical contact of the membranes or via membrane bound proteins. These connections are highly regulated and are dynamic in nature [4]. Among different organelles, one of the most studied interactions is between mitochondria and the endoplasmic reticulum (ER). The first evidence of this particular interaction was discovered in rat liver cells in 1952 [5] and 1956 [6,7] through electron microscopy. Later an elaborate and apparently unique specialization of the endoplasmic reticulum and a precise orientation with respect to the mitochondria has been described for the specific cell of the pseudo-branch gland in teleost [2,8]. The isolation of ER-mitochondria contacts was performed ~45 years ago by density gradient differential centrifugation from rat liver [9,10]. Vance subsequently coined the term mitochondria associated ER membranes [MAM] [11]. These functional sites are involved in lipid metabolism, calcium signaling, mitochondrial fission and fusion, ER stress, apoptosis and autophagy [3]. The distance between ER and mitochondria were estimated to be around 100 nm but later digital imaging microscopy and electron tomography studies proved that, they were at a distance of 10 to 25 nm during resting conditions, hence, they are referred to as ‘tethering of organelles’ [12,13]. However, during environmental stress, the contact sites become tighter (~10nm) [12]. The molecular foundation of this relationship is complex and involves not only ion homeostasis through the shuttling of calcium but also many structural and apoptotic proteins. The important proteins that are involved in the tethering are mitofusin (MFN), inositol triphosphate receptor (IP_3_R), voltage-dependent anion channel (VDAC), glucose-regulated protein 75 (Grp75), mitochondrial fission 1 protein (Fis1), B-cell receptor-associated protein 31 (BAP31), protein tyrosine phosphatase interacting protein 51 (PTPIP51) and vesicle-associated membrane protein-associated protein B (VAPB) [14]. Studies showed different mitochondrial proteins can regulate the ER stress and Unfolded Protein Response (UPR) pathways. The outer mitochondrial membrane GTPase mitofusin 2 (Mfn2) is known to regulate the shape of endoplasmic reticulum (ER) controlling ER-mitochondrial contacts. Interestingly deletion of Mfn2 causes activation of ER stress [15]. Recently a unique regulation of the ER stress through a 54-amino acid microprotein, PIGB opposite strand 1 (PIGBOS), has been demonstrated. This microprotein localizes to the mitochondrial outer membrane where it interacts with the ER protein CLCC1 at ER–mitochondria contact sites. Loss of PIGBIOS led to activation of UPR [16]. Alternately, a large-scale high throughput study on human cervical cancer cells showed that ER stress can regulate the translations of mitochondrial genes, specifically those involved in mitochondrial localized translation [17].

This review will primarily focus on the ER stress sensors and its relation to ER-mitochondrial communication, MAM proteins and its involvement in various cellular functions. A special effort has been taken to discuss recent insights/developments about the non-canonical role of ER stress sensors in different physiological processes beyond its canonical ER stress signaling.

## 2. ER Stress and ER Stress Sensors

Endoplasmic reticulum is an organelle that spans a large area in the cytoplasm in the shape of elongated tubules and flat discs. The presence of ribosomes on the ER makes it Rough ER (RER), while the absence makes it a smooth ER (SER). With the presence of ribosomes, one of the major functions of ER is synthesis, folding, maturation and degradation of secretory and transmembrane proteins. ER plays a crucial role in maintaining the cellular protein homeostasis or proteostasis. Before the export of proteins from ER to Golgi apparatus, the proteins undergo post-translational modifications and folding with the help of chaperons and folding enzymes/foldases such as Protein Disulphide Isomerases (PDI) present in the ER. Proteins such as calnexin and calreticulin act as a quality control for the proteins to ensure if they are properly folded [18].

Many exogenous and endogenous factors such as UV radiation, reactive oxygen species, hypoxia, protein mutations, lipid homeostasis, deletion of genes and nutrient starvation can cause accumulation of misfolded proteins resulting in ER stress. Also, the membrane phospholipid synthesis is important in maintaining the function of the organelles, hence impairment of phospholipid biosynthesis can upregulate the protein quality control pathway such as the UPR of ER or ER-associated protein degradation (ERAD) [19]. For instance, Inositol Requiring Enzyme 1 (IRE1) is activated in the absence of inositol in yeast, as it is essential in the phospholipid metabolism and is required by *INO1* gene which encodes inositol-3-phosphate, an enzyme of the phosphatidylinositol synthesis. When UPR occurs, the genes related to lipid biosynthesis are upregulated. In mammals, the spliced X-box binding protein 1 (xbp1) acts as a transcription factor and it subsequently activates the lipid synthesis genes. Direct evidence that lipids may activate the UPR independently was first provided by Promlek et al. (2011) in budding yeast [20]. Similarly, Volmer et al. showed membrane lipid saturation can activate UPR without the accumulation of misfolded proteins in mammalian cells [20,21]. The ER stress activation through gene mutation involved in lipid biosynthesis without the presence of any misfolded stress has been observed in *C elegans* also [22]. Surprisingly, it was noted by Shyu et al. 2019 that stress caused in the lipid bilayer (especially phosphatidylcholine and phosphatidylethanolamine) can induce ER stress in budding yeast and the reduction of UPR to restore the lipid homeostasis is seen in various human diseases, such as non-alcoholic fatty liver, diabetes, cardiac and muscle dystrophies. Major perturbations to PC or PE can prematurely degrade certain transmembrane proteins such as sarco/endoplasmic reticulum Calcium-ATPase (SERCA) ion pump, which leads to disturbance in the calcium homeostasis ultimately resulting in ER stress. Also, the Sec61 translocon on the ER gets degraded by Doa10 complex which does not allow the misfolded proteins to exit ER [19]. Eukaryotic organisms switch on the UPR of ER that initiates a cascade of cellular signaling to restore homeostasis and regular ER function [23].

**Figure 1:**
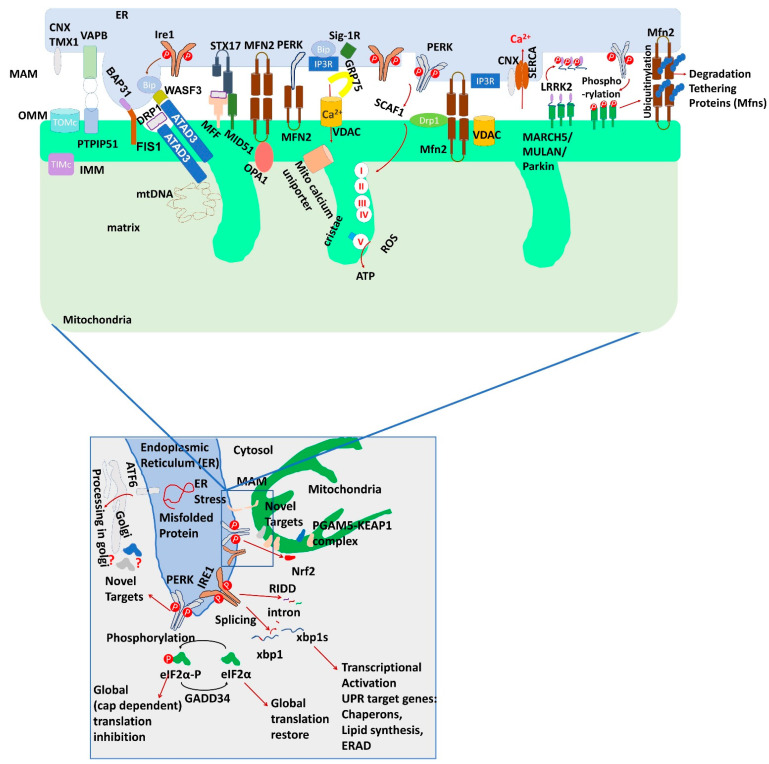
Endoplasmic reticulum (ER) mitochondrial contact: membrane dynamics, signaling and involvement of ER stress sensors. (**Top**), the schematic representation of ER-mitochondrial contact with key molecular contributors and their known functional interactions are summarized. MAM proteins are generally anchored either on the ER or outer mitochondria surface. Some MAM proteins like ATAD3A span from the inner mitochondrial membrane to ER -Mito contact site. Calcium transfer between ER-Mitochondria regulates through different channel proteins distributed throughout the MAM site. IP3R on the ER membrane transfers calcium from ER lumen to the mitochondria which is mediated by GRP75 and is taken up by VDAC at OMM and transferred to IMM via MCU. SERCA formulates the return of calcium to ER. SIGMA 1R protects IP3R from ERAD. TIMC/TOMC, protein translocase complexes of IMM and OMM mediating mitochondrial protein import. TMX-1 at MAM forms a functional complex with Calnexin (CXN) and SERCA to modulate calcium flux. VAPB an ER integral membrane protein and PTPIP51 an OMM protein interact with each other to regulate lipid transfer. MFN2 in ER and mitochondria/ MFN1 in mitochondria are tethering proteins which can also regulate calcium homeostasis. MAM proteins, LRRK and PERK control ubiquitination pathway dependent mitochondrial tethering process. MARCH5/MULAN/PARKIN modulates the structure of MAM through degradation via LRRK mediated MFN2 ubiquitinoylation. Mitochondrial dynamics are maintained by the fission fusion process. Successful mitochondrial fission requires coordination of ER resident STX17 with mitochondria bound Mff and MiD51 via DRP1 to initiate mitochondrial fission. Similarly, for fusion processes MFN2 and OPA1 interact with each other. ATAD3A forms a ternary complex involving ATAD3A, WASF3 and MAM associates GRP78 to regulate cell proliferation during metastasis. ATAD3 complex with cytosolic DRP1 controls mtDNA maintenance. PERK/eIF2α/ATF4 axis transcriptionally controls SC assembly factor 1 (SCAF1) levels to maintain the formation of respiratory supercomplexes. (**Bottom**), schematic representation of ER stress activation upon accumulation of misfolded proteins. UPR sensors PERK, IRE1α, ATF6, is activated due to the stress. Auto phosphorylated/Activated PERK phosphorylates the eIF2α that results in attenuation of global protein translation. GADD34 can dephosphorylate eIF2α making the translation attenuation transient. PERK can activate latent NRF2 in the cytoplasm to enhance the survival of cells. Phosphorylated cytosolic domain of IRE1α cleaves the introns from XBP1 mRNA which then encodes for XBP1s protein. It induces the expression of UPR target genes, chaperons, ERAD. IRE1 degrades a set of mRNAs in the ER termed IRE1-dependent decay of mRNAs (RIDD). Activated ATF6 is transported to the Golgi where it is proteolytically cleaved to an active form. Now it enters the nucleus to induce expression of UPR target genes. MAMs have been shown to be a hot spot for the transfer of stress signals from the ER to mitochondria specially during the loss of ER homeostasis. Details of all the abbreviations are at the end.

UPR of ER was first studied on yeast, where it is solely regulated by IRE1, while in mammals there are three major proteins involved in controlling ER stress response: IRE1, protein kinase RNA-like endoplasmic reticulum kinase (PERK), activating transcription factor 6 (ATF6) [23] (Figure 1, bottom). These ER stress sensors get activated upon the presence of misfolded proteins and their activation mechanism is still unclear [22,23]. If UPR fails to rescue the cellular protein homeostasis, the cells undergo apoptosis.

These sensors have an ER luminal domain, ER transmembrane domain and a cytosolic domain to pass on signals to other machinery involved in protein functions. Each of these sensors act independently to attenuate translation (in case of PERK) and induce expression of UPR target genes (IRE1, ATF6) [24]. When there are no stress conditions prevailing, these proteins remain bound to a heat shock protein 70 chaperon (Hsp70) called BiP. When misfolded proteins are detected, these sensors are released from BiP. This is followed by oligomerization of PERK and IRE1. Trans-auto-phosphorylate at their cytosolic domains activate their downstream signaling pathway. ATF6 is transported to the Golgi for its further actions [25].

IRE1(also known as ERN1, for ER to nucleus transducer 1) is a highly conserved UPR sensor protein with kinase and RNase activity [26] (Table 1). IRE1 and its downstream transcription factor HAC1/IRE2 (the yeast ortholog of the metazoan XBP1) were first identified as a factor required for growth in the medium deprived of inositol [26,27]. Inositol is an essential building block of yeast phospholipids. IRE1, a type 1 transmembrane protein, has both serine/threonine (S/T) kinase domain and an RNAse domain. In the search for the mammalian counterpart two groups independently identified two homologs of IRE1, named IRE1 alpha and IRE1 beta [28,29,30]. IRE1α is found ubiquitously while IRE1β is restricted to the intestinal epithelium. During ER stress the IRE1 is activated through direct or indirect activation by the misfolded proteins [31,32]. A subsequent oligomerization of the cytoplasmic domain of IRE1 leads to the activation of the RNase domain which splices introns from mRNA encoding the XBP1 [33]. The transcriptionally active XBP1 induces expression on genes such as Glucose regulated protein (GRP78), Protein disulphide isomerase (PDI) and other translocation proteins to regulate the protein folding during ER stress [34]. Also, it activates the expression of heat shock protein 40 kDa (DnaJ), p58, ER-resident molecule (ERdj4), ER degradation-enhancing α-mannosidase-like protein (EDEM) involved in ERAD and ER-to-Golgi transport components [25,35,36,37,38]. However, in the case of prolonged or unresolved ER stress, IRE1α cleaves various mRNAs localized in the ER through a process called Regulated IRE1α dependent decay (RIDD) [39,40].

ATF-6 is a leucine zipper protein, which is encoded by ATF6A for ATF6 alpha and ATF6B for ATF6 beta (Table 1). It is activated in the ER by misfolded proteins and exported to the Golgi where it gets cleaved by a protease; membrane-bound transcription factor peptidase, site1 (S1P). Approximately 400 amino acids from its N-terminal region are cleaved off and now it activates different UPR gene expression in the nucleus. Combining IRE1 and PERK activity, ATF6 also activates XBP1 and CHOP to enhance the UPR signaling pathway [25,41,42].

**Table 1:** Unfolded Protein Response (UPR) sensors from yeast to mammals.

			References
UPR proximal sensor	Yeast *Saccharomyces cerevisiae*	IRE1	[26,43]
	Metazoans *C. elegans*	IRE1	[26,44]
		Pek-1	
		atf6	
	Fly *Drosophila melanogaster*	IRE1	[45]
		PEK1	
		atf6	
	Mammals	IRE1α (ubiquitous) And IRE1ß (only in Gut)	[30,45,46]
		PERK/PEK	[46,47]
		ATF6α and ATF6ß	[48,49]
Downstream transducers of Proximal sensor	Yeast *Saccharomyces cerevisiae*	Hac1	[34,50]
	Metazoans *C. elegans*	XBP1	[51,52]
	Fly *Drosophila melanogaster*	xbp1	[42]
	Mammals	XBP1 eIF2α	[33,53]

PERK is a major sensor protein identified from rat pancreatic islets, which identifies the imbalances in the ER during the stress conditions and resolves it by reducing the overall translation. The activation of PERK during ER stress is still unclear but after its activation, it can selectively bind to the misfolded proteins and not the native proteins [23,25] (Table 1). The ubiquitously expressed PERK has luminal and cytosolic serine/threonine domains. The BiP removal from the luminal domain is involved in the oligomerization and trans-autophosphorylation step which results in the activation of PERK [25]. The activated PERK phosphorylates serine 51 (Ser 51) of the alpha subunit in the eukaryotic translation initiation factor 2 alpha (eIF2α). The phosphorylation of eIF2α results in the inhibition of eIF2B, thus it eventually reduces the initiation of the global translation process and the subsequent protein load in the ER. The phosphorylation also results in translation of mRNAs encoding for several factors such as activating transcription factor 4 (ATF4), BiP, GRP94, XBP1, ATF6. During ER stress, all the above-mentioned proteins are required to improve cellular homeostasis. Some of the PERK associated pathways that are involved in ER-Stress induced apoptosis are PERK/eIF2α/ATF4, PERK/CaN, PERK/eIF2α/TDAG51, PERK/eIF2α/IAP2 and PERK/NRF2 [53,54]. One of the main downstream targets of eIF2α-ATF4 is CCAAT/enhancer-binding protein homologous protein (CHOP). The promoter site of CHOP has binding sites for the UPR activators such as ATF4 and ATF6. CHOP induces apoptosis by activating pro-apoptotic factors such as death receptor 5 (DR5), Bim and telomere repeat binding factor 3 (TRB3) and inhibits anti-apoptotic factors such as BCL-2 [55]. The negative feedback loop in the mechanism is played by growth arrest and DNA damage-inducible 34 (GADD34), as it can dephosphorylate eIF2α and restart the protein synthesis [25].

## 3. Proteins at ER and Mitochondria Contact Site and Its Function

Mitochondria-endoplasmic reticulum (ER) contact sites are maintained by the physical interactions between the outer mitochondrial membrane (OMM) and the ER surface that run in parallel at a constant distance. The juxtaposition between these organelles controls several signaling pathways involving different kinds of proteins including ER stress sensor proteins (Figure 1, top). While MAM research progressed in the mammalian system very rapidly, the one in the yeast was studied less progressively [56]. Later in yeast few novel players have been identified that contributed to ER mitochondrial contact sites [57]. In yeast, MAM is termed as ER-mitochondria encounter structure (ERMES). The complex contains 4 proteins; Mdm10 and Mdm34, these are integral outer mitochondrial membrane (OMM) proteins, Mmm1, an integral ER membrane protein and Mdm12, a cytosolic protein [2,14,58]. These are involved in lipid exchange, calcium transfer, protein import, mitochondrial motility and genome maintenance [58]. Studies showed that mutations to this ERMES complex resulted in morphological defects in the mitochondria and altered lipid homeostasis [58].

The overall MAM protein complex in higher organisms is more advanced in their functionality than the ERMES complex and there are no exact homologs for the core proteins that are present in the yeast model. There are many proteins present in the MAM complex that attribute to the stability of the tethering model [2] (Table 2). Advancement of different microscopic techniques enables us to assess ER-mito contacts in a variety of live and fixed samples. In addition, assays based on co-localization markers with confocal or advanced super resolution microscopy provide sufficient resolution to detect the physiologically relevant interfaces between ER and mitochondria [59,60,61]. For example, structured illumination microscopy (SIM) helped to understand the role of DISC1, a MAM protein, in controlling mitochondrial dynamics and neuronal morphogenesis [62]. Moreover, new insights over VAPB-PTPIP51 interaction and ER-mitochondria associations during amyotrophic lateral sclerosis/frontotemporal degeneration (ALS/FTD) can be possible using these techniques [59]. Several other indirect approaches like fluorescence resonance energy transfer (FRET) based calcium sensors, yeast two hybrid screening, split luciferase assays have been instrumental in understanding the role of ER-mitochondrial dynamics [63,64,65]. Few new players in the MAM have recently been identified through proximity-based techniques (APEX coupled with mass-spectrometry). These approaches are effective not only to identify new players but also to confirm the existence of known MAM proteins as well [66,67]. Reticulon1A (RTN1A) promotes ER mitochondrial contacts and ribosome-binding protein 1 (RRBP1) as a binding partner for SYNJ2BP modulates ER-mitochondrial tethering have been identified from the two different APEX studies. Among other known MAM, these screen also found significant enrichment of an important MAM component, ATPase family AAA domain-containing protein 3 (ATAD3A) which is involved in several physiological processes including mitochondrial biogenesis, lipid biosynthesis and steroid biosynthesis, mtDNA maintenance, mitochondrial connectivity and mitophagy [68].Very recently, Kwak et al. (2020) introduced a split-pair system of BioID, CONTACT-ID, for identification of the MAM proteome in live cells [68,69]. This approach successfully identified 115 MAM-specific proteins. In addition, it also revealed membrane topologies of 85 integral membrane proteins. This study established FKBP8 (also known as FKBP38) as a new MAM protein. Previously this protein was known for its involvement in apoptosis under the stress conditions [70], Sonic hedgehog signaling [71] and mitophagy [72]. FKBP8 is an essential player in MAM formation and its expression level controls calcium Ion transport from ER to the Mitochondria. The various functions of different MAM proteins are being discussed further.

### 3.1. Lipid Homeostasis

The MAM is the main site for non-vesicular phospholipid and cholesterol transport and also the synthesis of certain intermediates during lipid synthesis. ER serves as the major site for membrane lipid synthesis while the mitochondrial membrane has high levels of phospholipids and low levels of sterols and sphingolipids. Mitochondria receives phosphatidylserine (PS) synthesized by the ER which is then converted to phosphatidylethanolamine (PE) by mitochondrial PS decarboxylase enzyme. This PE is transported back to ER for its conversion to phosphatidylcholine (PC), which is finally given to mitochondria for its membrane synthesis [73,74]. Proteins such as oxysterol-binding protein (OSBP)- related protein 5 and 8 (ORP5/8) is involved in the transport of phospholipid between two organelles. The exact mechanism of transport of PS to mitochondria from ER is not studied well but members of the oxysterol-binding protein (OSBP) family are involved. Along with this, cholesterol is being transferred from ER to mitochondria with the help of STARD1 protein [75]. This STARD1 protein binds to the OMM and transfers the cholesterol to the IMM where it is processed by CYP11A1 [76,77,78]. Abatement of function to any of these proteins will affect the interaction between mitochondria and ER and also the function of other proteins involved in tethering [2,3]. Lipid homeostasis has also been controlled by another MAM protein, ATAD3. It is a nuclear-encoded ATPases family protein specific to multicellular eukaryotes. The protein associated with diverse cellular activities and essential for normal mitochondrial–ER interactions. RNAi mediated suppression of ATAD3 homologue (ATAD-3) in *C. elegans* leads to reduction of intestinal fat content along with low mitochondrial activity [79]. Similarly, studies on human subjects also found deletion of ATAD3 clusters has adverse effects related to cerebellar dysfunction. At molecular level, ATAD3 deficiency led to aberrant mtDNA organization and is associated with elevated free cholesterol and increased expression of genes involved in cholesterol metabolism [80].

### 3.2. Calcium Transport

Calcium is an important intracellular messenger involved in various signaling mechanisms. ER and mitochondria are the hubs for calcium transfer and storage. Transfer of calcium between these two organelles are important for a cell’s life and death cycle and mitochondrial division [81]. The concentration of calcium ions is approximately 1mM inside the ER while it is near 100nM in the cytosolic region [82].

On the ER and OMM, IP_3_R and VDAC channels are present in close proximity and are regulated by the chaperon GRP75. From the ER calcium is released through IP3R and is transferred to the OMM via VDAC. Calcium eventually travels into the IMM through mitochondrial calcium uniporter (MCU). The site of calcium transfer is also regulated by the mitofusin in the mammalian cell. MFN 2 on the ER surface interacts with MFN 1 and MFN 2 on the mitochondrial membrane. When the calcium release from the ER is reduced, Sig-1R on the membrane of ER is released from BIP/GRP78 to stabilize IP3R and promote prolonged ER calcium release. Overexpression of any of these molecules leads to the excess calcium storage in mitochondria which can trigger apoptosis or can ignite the ER stress response mediated apoptosis [64,65]. 

Besides the above-mentioned MAM proteins, the functional characterization of an ER protein, PDZD8, has established its role in ER mitochondrial tethering. This protein is conserved in mammals and metazoans. In neurons, PDZD8 regulates the cytoplasmic Ca^2+^ dynamics through the balance between mitochondrial calcium uptake after synaptically induced Ca^2+^-release from ER [83]. Interestingly, a recent study found post-translational modifications by fatty acid can also play a crucial role in maintenance of calcium homeostasis via MAM proteins. Harada et al. 2020 demonstrated palmitoylated cytoskeleton-associated protein 4 (CKAP4) regulates mitochondrial functions through an interaction with VDAC2 at ER–mitochondria contact sites. Knockout of CKAP4 in various in vitro cell models and mice models showed morphological changes of mitochondrial and ER. In addition, palmitoylation of CKAP4 is essential in the formation of ER–mitochondria contact sites, Ca^2+^ influx into mitochondria, mitochondrial respiration and cancer cell proliferation. Hence, presence of MAM proteins is essential not only to maintain calcium homeostasis between ER and mitochondria but also regulate mitochondrial respiration and survival of cells.

### 3.3. Apoptosis

As a defense mechanism, when a cell is damaged by disease or during a stress response it undergoes apoptosis, while the exact signals triggering apoptosis in MAM is still unknown. The proteins at MAM directly or indirectly play a critical role in apoptosis. The excess release of calcium from the ER into the ER-Mitochondria interface can stimulate apoptosis. The calcium flux opens the mitochondrial permeability transition pore (MPTP with the help of cyclophilin D (CYPD), thus propagating Cytochrome C release, caspase cascade and, ultimately, apoptosis. The caspase targets BAP31 at the ER membrane and allows it to bind to FIS1 at the outer mitochondrial membrane (OMM), activating pro-caspase 8 [3,81,82,84]. PACS-2 an ER sorting protein is bound to Bid, a BH3-only protein family is important in ER-mitochondria coupling. During apoptosis by sustained ER stress PACS-2 releases Bid to mitochondria where it is cleaved by caspases to form activated truncated Bid (tBid), which promotes cytochrome C release and death. tBid can facilitate the accumulation of Bax/Bak in the OMM which are proapoptotic. The entire apoptotic signaling can be controlled by the presence of Bcl-2 family proteins in the ER as they bind to inositol-1,4,5-triphosphate (InsP3) receptors (IP_3_R) and inhibit calcium release. Several studies have shown that association with Bcl-2 family proteins, including BIM, helps stabilization of IRE1α oligomers to work against apoptosis during calcium overload [85,86,87]. In contrast, IRE1α as a member of MAM controls cell survival by splicing the Xbp1 mRNA [88,89,90]. In a neuronal cell line (SH-SY5Y), IRE1α knockdown causes cell death, through accelerated Ca^2+^ release from the ER. Induction of InsP3R increases ER Ca^2+^ release resulting in cell death due to prolonged mitochondrial Ca^2+^ accumulation and alterations in morphology (swelling and fragmentation) and function [91]. Anti-apoptotic miRNA is degraded by IRE1α. It results in increased mRNA and protein expression of TXNIP and caspase-2 [92,93]. ER stress sensor PERK has initially been identified as a crucial member at the MAM and transmits apoptotic signals from the ER to mitochondria [69]. The downstream target of PERK is CHOP, a proapoptotic gene that suppresses transcription of BCL-2 genes and stimulates the expression of Bax and Bim. CHOP can also increase Ero1α expression that favors calcium release from IP3R stimulating apoptosis [94]. 

### 3.4. Autophgy

In all eukaryotes, autophagy is a conserved process to maintain cellular homeostasis by auto digesting the unwanted or damaged parts of a cell. The molecular basis of the mechanism is well studied in yeast and around 30 autophagy-related genes (Atg) have been identified, out of which 18 are essential for autophagosome emergence. These Atg proteins are conserved from yeast to humans and they have similar functions [95].

Autophagosome is a structure that encompasses a damaged part of a cell and then fuse with the lysosome, this can originate from the ER-mitochondria contact sites called the isolation membrane. Autophagy is initiated by isolation membranes, which then fuses with the lysosomes to degrade the cellular components. But the origin of this isolation membrane was in debate, as they thought it can arise from either ER, mitochondria or plasma membrane [96,97]. Hamasaki et al., have performed imaging studies to show that autophagosomes form at the ER-mitochondria contact sites. The data revealed that the autophagosome marker ATG14, part of the PI3K complex that is essential for autophagosome formation, was found to be localized at the MAM during the starved condition of the cell [98,99] and is placed on the ER under normal conditions but during starvation, it diffuses through the ER membrane [100,101]. Electron tomographic studies showed that in presence of sufficient amino acid there was no localization of ATG14 on MAM but during starvation, accumulation of ATG14 was markedly observed at the MAM along with double FYVE domain-containing protein (DFCP1), a protein that can act as a platform for autophagosome formation [102]. Localization towards ER and mitochondria were seen throughout the process but the association with ER was stable while that with mitochondria was found only during amino acid starvation. ATG14 complex, as well as DFCP1, re-localizes to the MAM fractions during starvation. By these results, Axe et al. (2008) proved that ER gave the stage for autophagosome formation, while mitochondria have a role in providing components required for the same [102]. Live-cell imaging also showed that eventually when the expression of mitochondrial protein VDAC1 was increasing, the expression of ATG14 slowly decreased showing the end of autophagy. This again proves that the process of autophagy occurs at the MAM site [103]. Another ER protein known as STX17, it is known to be a Qa-SNARE protein, also localized at MAM during starvation but its exact function is not known yet [104]. It was also proved that STX17 was upstream of ATG14 and is responsible for the recruitment of ATG14 to MAM during any stressful conditions. 

To elucidate the relationship between ER stress, autophagy and apoptosis, Ji et al., performed a few experiments on osteosarcoma cell lines. Osteosarcoma is a tumor of the bone where the proliferating spindle cells produce immature bone [105]. The study established the role of ER stress sensor PERK in autophagy. Ekaterina Bobrovnikova-Marjon et al. found that PERK protein was readily detectable in all 7 carcinoma cell lines compared to non-transformed breast epithelial cell line. PERK promotes tumor growth and proliferation by limiting progression of cell cycle through oxidative DNA damage checkpoints [106]. It also helps the cells to adjust during hypoxic stress by altering angiogenesis [106,107]. This study, for the first time, showed PERK is highly expressed in osteosarcoma cells and it is involved in a protective mechanism such as autophagy. During ER stress the PERK activation is more and PERK- mediated autophagy is a protective function against apoptosis of the osteosarcoma cells. This is protective as the activated PERK may inhibit mTORC1 pathway and inhibit apoptosis by enhancing autophagy under stress conditions. But knockdown of PERK may enhance the ER stress in osteosarcoma cells and also switch on the mTORC1 pathway which will inhibit autophagy and increase cellular apoptosis. Loss of PERK function may inhibit autophagy and makes the cell sensitive to stress-causing apoptosis [108]. 

Mitophagy is a selective form of autophagy in which mitochondria are specifically degraded [109]. The PINK1/PARKIN pathway is the most well studied pathway of mitophagy. It has been found that PINK1 and BECN1 re-localize at mitochondria-associated membranes during mitophagy and promote the ER mitochondria tethering [109,110]. It is not surprising that later it was found few MAM proteins are actively involved in the mitophagy process. For example, FUNDC1 is a novel MAM protein required for hypoxia-induced mitophagy [111]. Numerous studies have convincingly demonstrated the crucial role of macro-autophagy and mitophagy in the commitment and differentiation of stem cells. MAM protein, Atad3a is involved in the maintenance of human progenitor cells via the PINK/PARKIN arm of mitophagy [111,112,113]. Deletion of Pink1 in Atad3a-deficient mice rescued mitophagy related defects which in turn resulted in the restoration of the progenitor and hematopoietic stem cell pools [111,112].

Evidence shows that ER stress plays a major role in neurodegenerative diseases, like Parkinson’s disease (PD). It is characterized by the accumulation of mutant alpha-synuclein protein at the ER and an overload of this causes ER stress which activates the IRE1, PERK or ATF6 pathway. The study by Yan et al. 2019 showed that the accumulation of toxic proteins triggers the IRE1 and brings autophagy-dependent neuron death in *Drosophila* model [114]. This marks the onset and progression of neurodegeneration in PD. This autophagy was mediated by the JNK pathway independent of XBP1 protein. While the presence of XBP1 offered neuroprotective effects upon dopaminergic neurons in PD [114,115].

### 3.5. Maintenance of Mitochondrial Dynamics and ER-Mitochondrial Physical Contact

Mitochondria are highly dynamic organelle and undergo constant structural reorganization for the maintenance of mitochondrial and cellular function by contributing to the proper distribution of mitochondria in response to the metabolic needs of the cell for ATP [116,117,118]). As de novo synthesis of mitochondria is not possible, thus mitochondria undergo fusion/fission events to enable proper distribution of mitochondria inside cells [119,120]. Mitochondrial outer membrane mediates mitochondrial fission by recruiting the master fission mediator Dynamin-related protein (DRP1, the yeast ortholog of it is DNM1L) from the cytosol to mitochondria [121,122,123]. DRP1 circumscribes the OMM as a helical oligomer and hydrolyses the GTP causing a conformational change in the oligomer that cleaves the membrane and triggers fission. Other proteins involved in this process are the mitochondrial fission factor (Mff) [124,125,126] and the mitochondrial dynamics proteins MiD49 and MiD51 (also known as MIEF2 and MIEF1, respectively) which are responsible for the recruitment of DRP1 to the OMM. Optic atrophy 1(OPA1), an inner mitochondrial protein regulates mitochondria morphology and energetics of mitochondria through mitochondrial fusion. It participates in the fusion process by maintaining the cristae structure [127,128,129,130]. Interestingly, along with the mentioned protein physical contact with ER plays a significant role. Voeltz research group identified a three-dimensional structure of ER-mitochondria in yeast using electron microscopy which revealed ER tubules were associated with mitochondria which formed the constriction sites. Due to these constriction sites, the diameter of the mitochondria reduced to 140 nm from 210 nm approximately enabling the fission process [131]. In both yeast and mammalian cells, the accumulation of these proteins is found in the site where ER tubules circumference the mitochondria. Thus, the juxtaposition between the endoplasmic reticulum (ER) and mitochondria is a common structural feature that facilitates the mitochondrial fission/fusion process.

### 3.6. Metabolism

Apart from protein synthesis and degradation, a variety of metabolic pathways, such as gluconeogenesis, glycogen synthesis and breakdown, membrane lipid synthesis and recycling, fat storage and hormone and drug metabolism are also controlled by ER. MAM proteins are also involved in regulation of the metabolic pathways. For example, the VDAC at the outer mitochondrial membrane interacts with IP3R through the molecular chaperone glucose-regulated protein 75, allowing Ca^2+^ transfer from the ER to mitochondria [103]. Recent studies demonstrated that MAM proteins play a significant role in the insulin signaling pathway. IP3R gets phosphorylated by Akt localized in MAM [132,133] and thus reducing Ca^2+^ release and preventing apoptosis [132,134]. mTORC2 can also be found in the MAM interface and its presence increases in response to growth factors stimulation [133]. Finally, tumor suppressor phosphatase and tensin homolog is also localized in MAM and sensitizes cells to apoptosis by countering Akt-mediated phosphorylation of IP3R [135]. 

It was further demonstrated that MAM integrity is required for insulin signaling in the liver [136]; UPR sensors like IRE1 and PERK are involved in AKT signaling events controlling mitochondrial structure and glucose metabolism. In glioma cells, PERK silencing inhibits growth during low glucose stress through partially blocking AKT activation and subsequent inhibition of Hexokinase II (HK2)’s mitochondria translocation [137]. Interestingly, (PKB/AKT-mTOR) signaling controls the dynamics of IRE1 deactivation by regulating ER-mitochondria physical contacts as well as the autophosphorylation state of IRE1 [138]. Metabolic regulation through different MAM proteins including IRE1 and PERK definitely demonstrated a complex regulation through organellar communication. Similarly, an interesting metabolic regulation has been observed in neuronal cells. Neuronal functions and maintenance are highly dependent on glucose metabolism. Specific glucose transporters circulate glucose across the blood-brain barrier to the neurons, where it is converted to pyruvate via glycolysis [139]. This pyruvate is further metabolized to acetyl-CoA via mitochondrial respiration. A minor part of glucose metabolism in neurons occurs via hexosamine biosynthesis pathway (HBP) where the proteins are added with N-acetylglucosamine, which can be sensitive to mild fluctuations of glucose metabolism [139,140]. Harg et al. demonstrated that activation of UPR has a considerable effect on glucose metabolism in human neuro-blastoma [141]. When there is increased UPR there is a reduction in the O-GlcNAc modified proteins, not due to the reduced amount of glucose in the cells but it is due to the direct effect of UPR on HBP along with a decrease in mitochondrial respiration. This reduction in mitochondrial respiration or ATP production is not due to a decrease in mitochondrial number or changes in its morphology, rather due to UPR activation. Through a systematic screening of UPR specific inhibitors Harg et al. for the first time, revealed the involvement of IRE1 in determining the overall metabolic state of the cell [141].

### 3.7. mtDNA Maintenance

Although mitochondria and the ER have an intimate functional relationship, there has been no evidence that they are inherited in a coordinated manner during mitosis until Liza Pon group for the first time demonstrated the mitochondrial inheritance in yeast through ER-mitochondria tethering. Swayne et al. showed a role for cortical Endoplasmic Reticulum (cER) in anchorage of mitochondria in the bud tip. Mmr1p which has a homology with tethering complex proteins can associate with mitochondria and ER and localizes to opposing surfaces of mitochondria and cER enabling the inheritance of the organelle in daughter cells [142]. Further, it has also found that the distribution of mitochondria and mitochondrial DNA in yeast are linked to ER associated mitochondrial division sites [143]. Later ER-mitochondria contacts also found to be involved in mtDNA synthesis with mitochondrial division in human cells [144]. Importantly, mtDNA integrity is also crucial in the context of neurodegeneration. ATAD3A, a MAM protein, interacts with Drp1 in striatal neurons derived from Huntington’s disease (HD) patient-iPSC cells. ATAD3A forms oligomers which bridge Drp1-mediated mitochondrial fragmentation and mtDNA instability, leading to impaired mitochondrial biogenesis and neurodegeneration [145].

## 4. Role of ER Stress Sensor Proteins in ER-Mitochondrial Communication and Beyond

### 4.1. IRE1

The role of IRE1 is extremely diverse due to its broad-spectrum role in various cellular processes; however, it has been majorly implicated in ER stress. Few recent studies have identified its new role specifically in the context of ER-mitochondrial communication. It has been found that the AKT-mTOR signaling axis modulates the dynamics of IRE1 RNAse activity by regulating ER-mitochondria contact. The study demonstrated a two-step mechanism of IRE1 attenuation. The auto-phosphorylation first initiates the termination of IRE1 RNAse activity but the complete cessation of RNAse activity only occurs if ER-mitochondria contacts are reformed after their initial uncoupling by ER stress [138]. IRE1 controls expression of GRP78 upon ER stress. During ER stress GRP78 plays a significant role in the stabilization of WASF3 on mitochondrial membranes. Interestingly another MAM protein ATAD3 forms a ternary complex involving ATAD3A, WASF3 and MAM associates GRP78. ATAD3A, acts as a crucial mediator to promote cell invasion in breast and colon cancer via regulating GPR78-mediated stabilization of WASF3 [146].

Besides its canonical role in ER stress several new reports establish involvement of IRE1 in different physiological processes. Interestingly a recent study on yeast identified iRE1/hac1 splicing pathway is essential for cellular adaptation upon diauxic shift via mitochondrial enlargement. They demonstrated IRE1 dependent increase of mitochondrial gene expression upon diauxic shift [147]. Similarly, in higher organisms, the non-canonical function of IRE1α also determines the mitochondrial calcium uptake. IRE1 α deficiency alters mitochondrial metabolism in vivo. Through mutagenesis analysis, Carreras-Sureda et al. successfully dissected out the housekeeping gene property of IRE1 α and uncovered a contribution of IRE1α to the maintenance of MAM composition and function even in the absence of ER stress. IRE1 α, itself, determines the distribution of inositol-1,4,5-trisphosphate receptors at MAMs. This study proposed a new model that establishes the fact that IRE1α operates as a scaffold that stabilizes InsP3Rs at MAMs [148]. Not only controlling ER-mitochondrial dynamics and mitochondrial calcium uptake IRE1α plays a very crucial role in terms of cell survivability. Mitochondrial ubiquitin ligase (MITOL/MARCH5) inhibits ER stress-induced apoptosis through ubiquitylation of IRE1α at the mitochondria-associated ER membrane (MAM). This is the first study that shows the regulation of IRE1α activity by ubiquitylation through a mitochondrial protein. MITOL promotes K63-linked chain ubiquitination of IRE1α and subsequently prevents hyper-oligomerization of IRE1alpha and regulates IRE1α -dependent decay (RIDD) [138,149]. Additionally, MITOL also ubiquitylates another MAM protein mitofusin 2 (Mfn2) [150]. It enhances the GTPase activity of Mfn2, resulting in the tethering between the ER and mitochondria rather than mitochondrial fusion (Sugiura et al., 2013). Interestingly, it is already known that Mfn2 can also modulate the UPR and mitochondrial function via repression of PERK, another ER stress sensor and a member of MAM [151]. All these indicate a complex interrelation of ER stress sensors in controlling ER mitochondrial communication beyond its canonical role.

### 4.2. PERK

Accumulation of misfolded proteins inside ER activates PERK through its dimerization and auto-phosphorylation. The serine/threonine (S/T) kinase PERK relieves folding pressure through eIF2α phosphorylation mediated translation shutdown and increases ER proteostasis mainly through the transcriptional activation of ATF4 [152]. Beyond this canonical role, PERK is also involved in various UPR-independent signaling functions. Cullinan et al. 2003 identified PERK mediated activation of nuclear factor-erythroid-2-related factor 2 (NRF2) transcription factor for cell survival [153]. Later Agostinis group demonstrated that PERK is absolutely essential in ER mitochondrial sites to convey apoptosis during ROS induced ER stress [87]. Later several other studies also establish that PERK facilitates the propagation of different signaling routes from the ER to juxtaposed mitochondria by tethering these organelles. Munoz et al. found repression of PERK can control mitochondrial function. Mfn2 is a novel PERK modulator and its deficiency causes mitochondrial dysfunction through sustained activation of PERK [151]. It was also observed that PERK-regulated translational attenuation reduces mitochondrial protein import through the degradation of the TIM23 subunit TIM17A [154]. The Mfn2 -PERK signaling might also be linked through microRNA mediated signaling. During ER stress, miR-106b-25 cluster is repressed by the Perk-dependent transcription factors Atf4 and Nrf2 [155]. Conversely, another independent study demonstrated that Mir106b mediated Mfn2 suppression is absolutely required for mitochondrial fusion via PKM2 [156]. Taken together it can be concluded that during ER stress while PERK leads to Mir106b repression it may subsequently modulate mitochondrial fission. A very recent study has shown that during ER stress there is a dynamic remodeling of mitochondrial morphology by promoting protective stress-induced mitochondrial hyper fusion (SIMH) through PERK by depleting Yme1l [157]. A recent study identified a direct link between the PERK mediated ubiquitination pathway and another MAM component, Leucine-rich repeat kinase 2 (LRRK2). It was found that LRRK2 regulates endoplasmic reticulum–mitochondrial tethering and mitochondrial bioenergetics. LRRK2 regulates the activities of E3 ubiquitin ligases MARCH5, MULAN and Parkin via kinase-dependent protein–protein interactions. When the Kinase-active LRRK2(G2019S) becomes dissociated from these ligases, it leads to their PERK-mediated phosphorylation and activation. PERK mediated phosphorylation of those ligases, in turn, induces ubiquitin-mediated degradation of ER–mitochondrial tethering proteins [158].

Not only PERK mediated ER mitochondrial connection but also PERK dependent ER plasma membrane connection is crucial in maintaining cellular homeostasis during ER stress. Using proximity-dependent biotin identification (BioID), Vliet et al. identified the actin-binding protein Filamin A (FLNA) as a key PERK interactor. Loss of PERK results in disturbed actin cytoskeleton and increased cortical F-actin [159].

Starvation or nutrient deprivation has a significant impact on mitochondrial morphology and enhances mitochondrial hyper fusion to balance mitochondrial bioenergetic efficiency [160]. Nutrient stress due to glucose starvation demands a cellular energetic shift from cytosolic glycolysis to mitochondrial oxidative phosphorylation (OXPHOS) system in order to maintain cellular growth and survival [161]. Moreover, nutrient starvation can also enable ER stress by disrupting protein folding and glycosylation in the ER. In a recent study, Balsa et al. demonstrated how ER communicates with OXOPHOS system to increase ATP production and promote proteostasis in the cell through a previously unknown mechanism controlled by PERK. The study found that PERK activation during ER stress and glucose deprivation stimulates mitochondrial bioenergetics through the formation of respiratory supercomplexes (SCs). PERK activation mediated increase in SCs elevates mitochondrial respiration in cells expressing PERK but not in PERK depleted cells. Additionally, PERK activation also resulted in changes in cristae formation and the formation of cristae is absolutely required in SC formation during nutrient and ER stress. PERK/eIF2α/ATF4 axis transcriptionally controls SC assembly factor 1 (SCAF1) levels to maintain the formation of respiratory supercomplexes. Interestingly, the PERK dependent mechanism can restore the defects in patients with complex I mutations, thus proving PERK activation can be future targets for mitochondrial diseases [162].

A direct link between PERK mediated control on insulin resistance has recently been demonstrated by Biddinger group [163]. This study again is an example of novel regulation in metabolic control through PERK. The authors found that trimethylamine N-oxide (TMAO) binds to PERK at physiologically relevant concentrations and selectively activates the PERK mediated induction of the transcription factor FoxO1, a key driver of metabolic disease. TMAO which is derived from gut microbiota is increased by insulin resistance and associated with several metabolic syndromes in humans. At pathologically relevant concentrations, TMAO binds and activates PERK and subsequently promotes hyperglycemia. Inhibition of TMAO synthesizing enzyme, flavin-containing monooxygenase 3 (FMO3), with 3,30 -diindolylmethane reduces PERK Activation and Insulin resistance in vivo suggesting a potential route of the therapeutic intervention for metabolic syndrome.

Brown adipose tissue (BAT) is one of the major tissues involved in thermogenesis and plays an important role in metabolic function that contributes to energy consumption (Cannon & Nedergaard, 2004). During brown adipose tissue differentiation, phosphorylation of PERK happens without any ER stress. This PERK phosphorylation induces transcriptional activation by GA-binding protein transcription factor α subunit (GABPα), which is required for mitochondrial inner membrane protein biogenesis. This study also demonstrated that p-PERK controls mitochondrial and thermogenic gene expression via transcriptional activation by GABPα and UCP1-mediated thermogenesis in vitro and in vivo [164]. 

### 4.3. ATF6

ATF6 is the third sensor that plays a significant role in activation of ER stress pathways. Unlike IRE1 and PERK, Atf6 is unique for its specialized activation process. Upon ER stress, the ATF6 traffics from the ER to the Golgi apparatus followed by a sequential cleavage. The cleaved active form acts as a transcription factor of various genes to restore ER homeostasis. Though it is not a MAM component, it is also involved in different non canonical processes controlling organelle homeostasis beyond ER stress. The protective role of Atf6 beyond the canonical role during ER stress has already been observed while overexpression of only ATF6 arm led to activation of protective remodeling of ER proteostasis pathways [165,166]. Stress independent activation of ATF6 transcription factor in various in vitro cellular models selectively reduces secretion and extracellular aggregation of destabilized amyloid disease-associated proteins. No significant changes have been observed after selective overexpression of ATF6 on the secretion of endogenous proteome [167,168].

Recently Burkewitz et al. 2020 demonstrated the loss of atf6 promotes longevity in C elegans. Interestingly, the life span extension is not through canonical proteostasis pathways but through the modulation of ER-mitochondrial calcium homeostasis. Atf-6 regulates the ER function and lifespan through its proximal mediator, calreticulin/crt-1. As a downstream effect, the ER calcium release further leads to changes in mitochondrial dynamics and bioenergetics [169]. Not only in *C elegans*, another study conducted by Wang et al. 2018 with human mesenchymal stem cells (hMSCs) also showed that ATF6 can control the aging through maintaining organelle homeostasis. Inactivation of ATF6 led to organelles’ dysfunction and accelerated cellular senescence, a process in which FOS functioned as one of the mediators [170]. Not only stem cells, Druelle et al. (2018) found significant changes in ER morphology during cellular senescence in normal human dermal fibroblasts (NHDFs). Senescent NHDFs also exhibited activation of UPR along with ER expansion. Knockdown of Atf6α inhibited ER expansion, the modification of senescence-associated cell shape and decreased senescence-associated β-galactosidase activity [171]. Besides this non canonical regulation, maintenance of mitochondrial biogenesis and function are also controlled during ER stress via Atf6 and PGC1α. In hepatoma cell line, expression of estrogen-related receptor gamma (ERRγ), a regulator of mitochondrial function, has been enhanced by ATF6α and PGC1α [172].

Earlier ATF6 got its attention when it was found to be extremely important for adaptive response during Ischemia/Reperfusion (I/R). The canonical ATF6-dependent ER stress response genes conferred protection from I/R damage in ex vivo isolated perfused heart preparations and maintained contractile function [173]. Further studies on transgenic mice with ATF6 fused to the mutant mouse estrogen receptor (MER) gradually established the idea that the function of ATF6 was much broader than the canonical ER stress response [174]. Another subsequent study using the same mouse model determined ATF6 specific regulation of particular microRNA that can control the activity of ER luminal calcium-binding protein, calreticulin (Belmont et al., 2012). Overexpression of the active ATF6 transcription factor in the heart also has been shown to improve cardiac performance in mouse models of ischemic heart disease, through a mechanism involving ATF6-dependent regulation of the antioxidant gene, catalase [175]. Very recently using conditionally deleted cardiac myocyte specific ATF6 knockout mouse (ATF6 cKO) showed exacerbated myocardial damage in comparison to wild type (Blackwood et al., 2019b). The study also found ATF6 was required to induce the expression of a small GTP-binding protein, Rheb, and, thus, control the mTORC1-dependent growth in pathological hypertrophy. Similarly, another tissue specific role of ATF6 has been observed in the liver. Insulin sensitivity has been improved significantly after overexpression of the active ATF6α transcription factor in the liver of obese mice [176]. Gain- and loss-of-function studies in mouse livers and in hepatocytes also identify ATF6α mediated increase of hepatic fatty acid oxidation through coactivation of PPARα to attenuate hepatic steatosis [177]. In the mouse studies ATF6 denotes the Atf6α which is expressed in all cell types.

The control of ER function and mitochondrial function have historically been studied as separate entities. But recent studies accumulated enough evidence of overlapping metabolic and signaling pathways for coordinated action of ER and mitochondria. Although UPR and its sensors (IRE1, PERK and ATF6) have classically been linked to ER stress, increasing evidence suggests that the sensors have various non-canonical functions in various cellular processes beyond secretory pathway surveillance during ER stress. Specifically, IRE1 and PERK as a member of MAM are directly involved in various signaling events that establish coordinated function with mitochondria and other cellular organelle.

## 5. MAM Proteins (IRE1 and PERK) in Neuronal Disease

Neurodegenerative diseases are characterized by the depletion of neuronal cells and damage to the axons, dendrites and synaptic buttons that ultimately lead to neuronal death. Parkinson’s disease (PD), Alzheimer’s disease (AD), Hereditary spastic paraplegias (HSP), Frontotemporal dementia (FTD), Amyotrophic lateral sclerosis (ALS) and Charcot marie tooth (CMT) are some of the major NDD [178]. Association between mitochondria-associated ER-membrane (MAM) and neurodegenerative disorders (NDD) are an important area of research. Deregulation in certain proteins present at the MAM can alter the function of neurons, its response to stress stimuli or influence the neuroinflammatory response of NDD [179]. Some of the major roles of MAM are associated with calcium homeostasis, mitochondrial dynamics and autophagy. In NDD all the above-mentioned functions are impaired as many proteins involved in NDD reside in MAM [99]. The proteins involved in AD, dementia, motor neuron disease, neuropathy, schizophrenia and chorea are abundantly present in MAM and deregulation in any of the proteins affect the cross-talk between ER and mitochondria [180].

AD is most commonly seen in elderly people and is associated with the formation of intracellular tangles of hyperphosphorylated tau and formation of plaques by beta-amyloid peptides, due to the cleavage of amyloid precursor proteins by beta and gamma-secretase which has a core dimer of Presenilin 1 and 2 (PS1, PS2) [181]. It is found that PS1 and PS2 are enriched at MAM and affect the communication between ER and mitochondria. When the Ps 1 or 2 are knocked out, the calcium transfer between ER and mitochondria and phospholipid synthesis is increased. This phospholipid increase can act as an important marker for finding AD [182,183]. Also, dysregulation of phosphofurin-acidic cluster sorting protein and sigma1R in MAM is a hallmark for AD, as sigma 1R is the master regulator for Tau phosphorylation. When the lipid bilayer of MAM has disrupted it results in the abnormal production of beta-amyloid peptides leading to AD [74].

One of the hypotheses on AD pathology so far is dependent on the abnormal structure of mitochondria and impaired function of MAM. But apart from proteins present at the MAM, AD brain also shows an increase in chronic UPR activation by increasing the levels of Grp78, phosphorylated PERK (pPERK), phosphorylated IRE1α, phosphorylated eIF2α (p-eIF2α) and ATF4 [184,185]. There are multiple studies that establish the intricate link between IRE1α and its role in AD [186]. In AD patients, accumulation of IRE1 is seen in the hippocampus region resulting in granulovacuolar degeneration in neurons [185]. The phosphorylated IRE1 activates XBP1 splicing which is found to be higher in the cortical areas of patients with AD and not in age matched normal subjects [187]. Also, the 116C/G polymorphism of XBP1 is a major risk factor for AD [188]. The overexpression of this downregulated ryanodine receptor 3 expression results in calcium dyshomeostasis in the cytosol leading to beta-amyloid toxicity [189]. Hence, targeting IRE1 can result in reduced amyloid deposits, improved cognitive and synaptic function and attenuated astrogliosis [190]. In the case of PERK, activation of ATF4 increases the pro-apoptotic cascades leading to apoptotic cell death in AD [191]. UPR activation also results in the accumulation of granulo-vacuolar degenerating bodies (GVDs), an autophagic vacuole produced by the smooth ER which encapsulates ubiquitinated protein for autophagic destruction [192,193]. The tau protein is localized along with GVDs and enhances the ER stress and intensifies the PERK’s immunoreactivity [194]. Studies on tau and its action on PERK are not well studied but PERK’s action over tau has been studied by several groups. pPERK activates GSK3 beta (implicated in tauopathy), which in turn activates the caspases leading to cleavage in tau. This caspase cleaved tau accumulates in the brain and is an indicator for pre-tangle pathology in AD [188,189,195,196]. Recently, direct PERK activation has been explored for neuroprotection by compound CCT020312 in models of tauopathies [197].

PD is characterized by loss of dopaminergic neurons in the substantia nigra pars compacta and the presence of Lewy bodies, cytosolic inclusions largely composed of alpha-synuclein. This alpha-synuclein is found in the MAM and its mutant variety affects the morphology of MAM and its calcium transfer between the organelles [198]. PTEN-induced kinase 1 encoded by the gene PINK1 is located at the mitochondrial outer membrane acting as a quality control protein and allows the binding of parkin protein to the dysfunctional mitochondria and causes autophagy of the same [199]. If the gene is silenced in the case of PD it reduces the contact between ER and mitochondria and deducts its biological functions [199,200,201]. 

During PD in dopaminergic neurons, there is also an increase in the levels of pPERK and p-eIF2α [202]. The major protein of PD, synuclein has no effect on UPR and is only associated with MAM and its dysfunction, hence UPR is a therapeutic target in this case [23,203]. A recent study on *Drosophila* exposed to heat-induced ER stress showed that PERK mediated attenuation of protein synthesis resulted in neuronal survival during heat stress. When the PERK-eIF2α pathway was impaired, there was dopaminergic neuron loss and decreased locomotor activity [203]. 

ER stress is an important contributor to the pathological implication in PD and accumulation of IRE1 is a major cause of this [204]. Ablation of XBP1 can be neuroprotective to dopaminergic neurons, this can be achieved by increasing the expression of UPR chaperons such as calreticulin and disulphide isomerase ERp72 in substantia nigra pars compacta [115,204]. A study on Drosophila elucidated that a mild ER stress-activated IRE1 pathway and not the CHOP regulated apoptosis in PD condition. While if we knock down the expression of XBP1 during PD, the CHOP pathway may get activated and result in neurodegeneration [205]. Targeting this pathway can promote dopaminergic neurons and neural stem cell survival and improves the symptoms of PD [205,206]. Recently a study using the Drosophila model showed that XBP1 independent overexpression of IRE1 resulted in autophagy-dependent neuron death via JNK pathway, [115]. The Leucine-rich repeat kinase 2 (LRRK2) gene is one of the most important genes in familial Parkinson’s disease (PD). Toyofuku et al. (2020) identified the role of LRRK2 in the ER–mitochondrial tethering, which is essential for mitochondrial bioenergetics. LRRK2 regulates endoplasmic reticulum–mitochondrial tethering through the PERK-mediated ubiquitination pathway [158].

Diseases affecting the peripheral nervous system by affecting the motor neurons are also linked to dysfunction in MAM. It includes Hereditary spastic paraplegias (HSP), a term used to describe a group of inherited NDD characterized by progressive weakness and spasticity [207]. HSP is majorly caused by an alteration in the SPAST gene encoding spastin, which is a microtubule-serving ATPase that has a role in axonal transportation and cytoskeleton organization. Along with spastin atlastin-1 and receptor expression enhancing protein 1 (REEP-1) it coordinates the connection between ER and microtubules [208]. When SPAST gene gets deleted, it causes a malfunction in the ER structure and function, resulting in defective MAM [64]. CMT is very complex and causes defects to the peripheral nervous system. Mfn2 was the first protein found in the MAM region and it is found to be mutated in the case of CMT type 2a. The mutations in Mfn2 are scattered in its C and N terminal both of which are extended into the cytosol, hence it causes defects in the tethering process of ER and mitochondria [209]. 

ALS is a late-onset fatal NDD, occurring sporadically and causes loss of motor neurons and muscle weakness [210]. The genes associated with ALS are antioxidant protein superoxide dismutase1(SOD1), TAR-DNA binding protein 43 (TDP-43), vesicle-associated membrane protein-associated protein B (VAPB) and valosin containing protein [211]. VABP is a resident at MAM and regulates the normal functioning of the same. When VAPD and TDP-43 are mutated the MAM is dysregulated by altered calcium homeostasis and impaired mitochondrial axonal transport. Sigma receptor 1 is located at MAM and is highly mutated during ALS and affects the lipid metabolism [212]. In the case of ALS, mutant forms of TDP-43 and SOD1 are upregulated during PERK activation resulting in the formation of stress granules [23]. They are aggregates of mRNAs, ribosomal subunits and other proteins formed due to the attenuation of translation by prolonged phosphorylation of eIF2 alpha [213,214]. ER-mitochondria interactions are perturbed by TDP-43, a protein pathologically linked to amyotrophic lateral sclerosis and fronto-temporal dementia. The perturbation is associated with disruption to the VAPB–PTPIP51 interaction and cellular Ca^2+^ homeostasis [215]. PERK regulates the development of ALS via several routes, for example, by affecting SOD1. SOD1 is an enzyme with three different isoforms. Cytosolic and mitochondrial intermembrane space-localized isoforms bind to copper or zinc ions and perform dismutation of free superoxide radicals. The SOD1 mutant form interacts with Bip and increases ER stress and mitochondrial oxidative phosphorylation. It also activates PERK and its downstream pathway. Higher expressions of p-eIF2α and ATF4 were observed in transgenic ALS (SOD1^G93A^) mouse spinal cords [216]. Several other studies also indicated the potential link between mutant and familial forms of ALS [217,218]. Though the actual cause of ALS (90% sporadic ALS) is elusive but only with respect to PERK the regulation is found to be at different levels. For example, haploinsufficiency of PERK significantly accelerates disease onset and shortens the survival of G85R mutant SOD1 transgenic mice which is phenotypically a model of familial ALS [219,220]. Moreover, PERK mediated induction of expression of two other chaperones, BIP and PDI, has been observed in SOD1 mutant lines. In addition, studies on ALS patients and mouse models demonstrated the PERK interaction of with Sigma1R, another MAM protein that is associated with ALS/FTD [221], suggesting the involvement of MAM remodeling in the development of ALS.

Huntington’s disease (HD) is caused due to the expansion in CAG repeat in the exon 1 of the huntingtin gene, which translates into a polyglutamine (polyQ) tract in the huntingtin (Htt) protein [222]. The expansion (above 35 glutamine residues) of the polyQ repeats causes mutant Htt (mHtt) to aggregate in HD tissues [223]. It damages the striatum and cerebral cortex but the exact pathway or mechanism is still unclear [224]. There are various types of Huntington’s disease-like syndromes (HDL) such as HDL- 2, 3 and 4 each showing different repeat expansion in different sets of chromosomes [225]. A mutant form of Huntingtin protein induces ER stress and levels of p-eIF2α are high in mutant Huntingtin [226]. Overexpression of mutant Huntington increases the PERK pathway and also increases the levels of Grp78 but if we target and reduce the phosphorylation at eIF2α, the toxicity caused by PERK can be reversed [226,227]. In the case of HD, the presence of p-IRE1 has been detected in striatal tissues of HD patients and it stimulates the aggregation of Huntingtin protein along with TRAF 2 (TNF- receptor-associated factor 2) which in turn induces neuronal cell death. Few mouse model studies showed the presence of XBP1 in the striatum of HD patients while it was absent in the cerebellum and cortex of the same subjects [228,229,230]. Evidence shows that ablation of XBP1 can reduce the accumulation of mutant proteins by increasing the expression of FoxO1 dependent autophagy pathway and enhancing motor neuron performance and neuronal survival (Wei et al. 2016). Another recent study also confirms that the sensitivity of the striatal neurons towards pathogenic HD is through the PERK-eIF2α pathway [231]. This study also demonstrated that the PERK pathway is strongly downregulated in striatal neurons compared to other cell types and brain regions in WT mice. In HD condition it has been upregulated. In the follow-up study, the Lederkremer group very recently showed PERK activator MK-28 can successfully reduce toxicity and extend survival in Huntington’s disease models [232]. 

Mutant ATAD3A contributes to neurodegenerative diseases affecting both peripheral and central nervous systems [233]. In conditions such as HD, ATAD3A exhibits a gain of function through oligomerization which causes mitochondrial fragmentation and impaired mitochondrial biogenesis leading to neurodegeneration. In HD cells, ATAD3A interacts with DRP1 (mitochondrial fission GTPase) and induces mitochondrial fragmentation. By blocking this interaction, mitochondrial fragmentation and mtDNA damage can be suppressed which ultimately reduces HD pathology [145]. 

Progressive supranuclear palsy (PSP) is an NDD with several phenotypes and also a type of tauopathy formed due to the accumulation of defective tau proteins with four MT-binding repeats in regions such as basal ganglia, diencephalon, brainstem and cerebellum [234]. Activation of PERK is seen in PSP human tissues and pPERK increases the level of tau proteins [235]. Eukaryotic translation initiation factor 2 alpha kinase 3 (EIF2AK3) encodes PERK. A GWAS study by Schellenberg and colleagues identified EIF2AK3 as a risk factor for PSP [236]. Several SNPs were found in coding as well as non-coding regions of EIF2AK3. These various SNPs in the EIF2AK3 genes can acts as a novel therapeutic target [235,236]. We have summarized a table (Table 2) describing the major MAM proteins categorized according to their functional role [237,238,239,240,241,242,243,244,245,246,247,248,249,250,251,252,253,254,255].

## 6. Conclusions

Conclusively, MAM site is a hot spot for the transfer of signals between the ER and mitochondria. Along with other MAM proteins, IRE1 and PERK, two membrane bound ER stress sensors, regulate signaling events not only during stress but also in several other physiological processes. Undoubtedly, new studies have described non-canonical roles of ER stress sensor beyond its stress response signaling. As a MAM component, both can interact with several other mam proteins to convey signaling that controls cell survivals. IRE1α regulation of RNAs (mRNAs and miRNAs) is an elegant process that manages to handle multiple cellular functions, apart from its decisive role in ER stress. Similarly, PERK has diverse regulation from interaction with ER-mitochondria to ER plasma membrane in controlling cellular bioenergetics to calcium transport maintenance. Though ATF6 is not present at MAM, it has important non-canonical role beyond ER stress. Therefore, a thorough understanding of these protein molecules and their involvement in diverse cellular events are needed to understand the complicated roles of these sensors beyond ER stress. Valid questions include: Apart from misfolded proteins, how cellular homeostasis mechanisms can be coupled with activation/inactivation of IRE1α and PERK? How different organelle contact influences cellular homeostasis through these ER stress sensors? Does PERK have additional targets beyond eIF2alpha like NRF2 controlling ER mitochondrial communication? An enhanced understanding of these two players can lead to targeted therapeutic interventions as these two molecules are highly involved in the development of different diseases including neurodegenerative disease through different pathways. 

## Figures and Tables

**Table 2:** Major mitochondria-associated ER-membrane (MAM) proteins and its functions.

S.no	Role	Protein	Function	Reference
1	Mitochondrial dynamics and morphology	VAPB	Regulates ER-Mitochondria interactions and also binds to PTPIP51 in mitochondria to regulate different functions at MAM.	[59]
		DISC1	Regulates mitochondrial dynamics by regulating calcium transfer.	[62]
		CKAP4	Regulates mitochondrial respiration, survival and calcium influx.	[81,82]
		Opa 1	Tether and fuse the inner membrane of the organelle.	[84]
		Fis1	Binds to Drp1 during fission and also alters the morphology of mitochondria based on ATP or calcium availability.	[84]
		PERK	Essential as ER mitochondrial contact to convey apoptosis on ROS based ER stress.	[87]
		MFN 1 and MFN 2	Involved in fusion and tethering to other organelles.	[237]
		Drp 1	Involved in fission.	[238]
		Mff and MIEF 1/2	Works together and recruits Drp1 to mitochondria during the fission process.	[238]
		PACS 2	Key regulator for the communication between ER and mitochondria.	[239]
2	Calcium homeostasis	PDZ8	Regulates cytoplasmic calcium dynamics.	[83]
		VDAC1	Gatekeeper for the transport of calcium in and out of the mitochondrial membrane.	[241]
		Sig-1R	Stabilizes IP3R and promotes prolonged ER calcium release.	[241]
		HSPA5	Regulator for calcium signaling and cell survival at MAM.	[240]
		PSEN1/2	Regulate calcium homeostasis at MAM.	[242]
		RyR2	Calcium-binding proteins located at MAM.	[243]
		IP3R	Mediates release of calcium from ER into the cytosol.	[244]
		ITPR1/3	Mediate release of calcium from ER, transport calcium to mitochondria by interacting with VDAC, calcium overload at ER lumen can initiate apoptosis via ITPR.	[245]
3	Lipid homeostasis	SERCA 1	Involved in intracellular mitochondrial trafficking and phosphatidylglycerol remodelling.	[19]
		ATAD3A	Involved in lipid and sterol transport.	[68,69]
		ACAT	Helps in the conversion of free cholesterol into cholesterol esters at MAM.	[242]
		STARD1	Important for the synthesis of steroids at the mitochondrial inner membrane interacts with VDAC and transports cholesterol to mitochondria.	[244]
		ERLIN1/2	Maintain cholesterol homeostasis by selective binding and also involved in ERAD.	[246]
		CAV 1	Act as lipid raft scaffolds and cholesterol binding domain.	[247]
		Sig 1- R	Chaperone involved in lipid synthesis and transport at MAM.	[248]
		PEMT	Catalyzes the methylation for phosphatidylethanolamine to phosphatidylcholine.	[249]
		ORP5/8	Controls transport of phospholipid at MAM.	[250]
		ACSL 4	Converts long-chain fatty acids into their active form, involved in intracellular lipid storage and transports cholesterol from ER to mitochondria.	[251]
4	Apoptosis, Autophagy and Mitophagy	CHOP	Induces apoptosis by activating death receptor 5 (DR5), BIM and telomere repeat binding factor 3 (TRB3) and inhibits anti apoptotic factors such as BCL-2.	[41]
		FKBP8	Controls Apoptosis, mitophagy via calcium level regulation.	[70]
		Cyclophilin D	Regulates the release of cytochrome C and caspases from mitochondria by opening the mitochondrial permeability transition pore (MTTP).	[84]
		BAP+FIS1	Interact with each other to activate pro caspase 8 and induce apoptosis.	[84]
		STX17	Involved in mitophagy during hypoxic conditions or other cellular stress conditions.	[104]
		PARK 2	It is a pro autophagic protein which induces ubiquitination and proteasomal degradation at OMM. It inhibits fusion and induces mitophagy.	[110]
		PINK 1	Act as a quality control protein to mitochondria, helps in tethering of MAM and induces mitophagy in stressful conditions.	[110]
		FUNDC 1	Involved in mitophagy during hypoxic conditions or other cellular stress conditions.	[111]
		AKT/mTOR	Proteins of this pathway are localized at MAM and at mild ER stress, they are triggered to induce autophagy as a protective mechanism.	[133]
		ATG 14	Regulates the formation of autophagosomes and acts as an autophagosome marker.	[252]
5	Metabolism	PIGBOS	Interacts with CLCC1 and regulates UPR and cell survival.	[16]
		IRE1	IRE1 signaling induces a reduction in glucose metabolism in neurons.	[141]
		PERK	PERK is required for the hyperglycemia induced by TMAO	[164]
		Akt	It reduces calcium release by phosphorylating Ip3 and reduces apoptosis. This phosphorylation is regulated by the glucose level and it maintains insulin homeostasis.	[254]
		PP2A	It regulates the structure and function of MAM by the amount of glucose present. High glucose content reduces PP2A thus it gets activated and phosphorylates downstream MAM proteins.	[255]
6	MAM tethers	Reticulon 1A (RTNIA) Ribose-binding protein 1 (RRBP1) + Binding protein of SYNJ2B	Increase the extent of MAM contacts.	[66,67]

## Data Availability

Not applicable.

## Abbreviations

ER—Endoplasmic reticulum; Mito—Mitochondria; MAM—Mitochondria associated; ER membrane; MFN—Mitofusin; IP3R—Inositol triphosphate receptor; VDAC—Voltage-dependent anion channel; GRP75/78—Glucose-regulated protein 75/78; PTPIP51—Protein tyrosine phosphatase interacting protein 51; VAPB—Vesicle-associated membrane protein-associated protein B; SERCA—Sarco/endoplasmic reticulum Calcium-ATPase; IRE1—Inositol requiring enzyme 1; PERK—Protein kinase RNA-like endoplasmic reticulum kinase; ATF6/4—Activating transcription factor 6/4; OMM—Outer mitochondrial membrane; IMM—Inner mitochondrial membrane; HSP 70—Heat shock protein 70; GADD34—Growth arrest and DNA damage-inducible 34; ATAD3A—ATPase family AAA domain-containing protein 3; DRP1—Dynamin-1-like protein; MFF—Mitochondrial fission factor; Sig- 1R—Sigma-1 receptor; STX17—Syntaxin 17; MITOL/MARCH—Mitochondrial ubiquitin ligase/Membrane-associated RING-CH E3 ubiquitin ligase; CXN—Calnexin; ERAD—ER-associated degradation; TIMC/TOMC—Protein translocase complexes of IMM and OMM mediating mitochondrial protein import; TMX1—Thioredoxin Related Transmembrane Protein 1; MiD51—Mitochondrial dynamic protein MID51; SCAF1—Super complex assembly factor 1; RIDD—IRE1α-dependent decay; XBP1—X-box binding protein 1; eIF2α—Eukaryotic Initiation Factor 2; LRRK2—Leucine-rich repeat kinase 2.
